# A Fragment of Collagen Type VI alpha-3 chain is Elevated in Serum from Patients with Gastrointestinal Disorders

**DOI:** 10.1038/s41598-020-62474-1

**Published:** 2020-04-03

**Authors:** Signe Holm Nielsen, Joachim Høg Mortensen, Nicholas Willumsen, Daniel Guldager Kring Rasmussen, Ditte J. Mogensen, Antonio Di Sabatino, Giuseppe Mazza, Lars Nannestad Jørgensen, Paolo Giuffrida, Massimo Pinzani, Lone Klinge, Jens Kjeldsen, Diana Julie Leeming, Morten Asser Karsdal, Federica Genovese

**Affiliations:** 1 Nordic Bioscience, Biomarkers and Research, Herlev, Denmark; 2 Department of Biotechnology and Biomedicine, Technical University of Denmark, Kgs. Lyngby, Denmark; 3 Department of Gastroenterology, University of Odense, Odense, Denmark; 4 First Department of Internal Medicine, San Matteo Hospital Foundation, University of Pavia, Pavia, Italy; 5 University College of London, Institute for Liver and Digestive Health, London, UK; 6 Digestive Disease Center, Bispebjerg Hospital, University of Copenhagen, Copenhagen, Denmark

**Keywords:** Enzymes, Assay systems

## Abstract

Extracellular matrix (ECM) remodeling is a hallmark of the pathology of gastrointestinal disorders. Collagen type VI (COL6) is produced by fibroblasts, and the COL6 α3-chain has shown to be elevated in patients with ulcerative colitis (UC), Crohn’s disease (CD) and colorectal cancer (CRC). Measuring COL6α3 in serum may therefore have potential as a biomarker for gastrointestinal disorders. The aims of this study were to develop and validate a competitive ELISA targeting a specific neo-epitope of COL6α3 and evaluate its associations with the gastrointestinal disorders UC, CD and CRC, in comparison to healthy controls. A monoclonal antibody was raised against a matrix metalloproteinase-2 and -9 specific cleavage site of COL6α3 (C6Mα3) and employed in a competitive enzyme-linked immunosorbent assay (ELISA). The assay was developed and technically validated. Levels of C6Mα3 were measured in serum from patients with UC (n = 58), CD (n = 44) and CRC (n = 39) and compared to healthy controls (n = 32). The levels of C6Mα3 were elevated in patients with UC, CD and CRC patients compared to healthy controls (all p < 0.0001). The area under the receiver operating characteristics (AUROC) curve for separation of patients with UC from healthy controls was 0.972 (95% CI: 0.925–1.020, p < 0.0001), with CD from healthy controls was 0.947 (95% CI: 0.885–1.009, p < 0.0001) and with CRC from healthy controls was 0.890 (95% CI: 0.809–0.972, p < 0.0001). We developed a technically robust assay targeting a fragment of COL6, which was elevated in serum from patients with UC, CD and CRC.

## Introduction

Inflammatory bowel diseases (IBD) are a group of idiopathic gastrointestinal chronic inflammatory conditions, encompassing ulcerative colitis (UC) and Crohn’s disease (CD), characterized by a dysregulated excessive immune response and tissue damage affecting the mucosa and submucosa of the gastrointestinal tract^1–5^. The extracellular matrix (ECM) is responsible for maintaining tissue architecture in the gastrointestinal tract, and plays an important part of the pathophysiology of UC, CD and colon cancer (CRC)^4,6^. Here an imbalanced ECM turnover may cause abnormal tissue function. Novel biomarkers reflecting altered ECM remodeling may therefore be an important tool for evaluating pathologically relevant disruptions in intestinal tissue.

One of the components maintaining ECM architecture by binding to different components in the ECM is collagen type VI (COL6)^7,8^. COL6 is characterized as a beaded filament collagen found in the interface between the basement membrane and interstitial matrix, where it forms a microfibrillar network. COL VI is present in various tissues, including the gastrointestinal tract. In cancer and IBD, the turnover of COL6 is altered due to an accumulation of fibroblasts and increased matrix metalloprotease (MMP) activity^9–13^.

While six different alpha chains of COL6 have been described (COL6α1–COL6α6), the COL6α3 chain has been found to be particularly relevant for CRC. COL6α3 affects tumorigenesis by providing pro-tumorigenic signals, but it has also been shown to have promising diagnostic and prognostic biomarker potential^14–19^.

In IBD COL6α1, COL6α2 and COL6α3 chains are elevated in the intestinal tissue in patients with CD and UC, and COL6 has been demonstrated to be involved in the integrity of the epithelial barrier^20,21^. It has previously been demonstrated that COL6 turnover was elevated in luminal CD and stricturing CD compared to fistulizing CD^22^.

Based on the function of COL6 and MMPs in cancer and IBD, we hypothesized that a specific MMP-generated COL6α3 fragment could function as a biomarker in gastrointestinal disorders.

The aim of this study was to develop and validate a competitive ELISA targeting a neo-epitope of COL6α3 and evaluate its associations with the gastrointestinal disorders UC, CD and CRC, in comparison to healthy controls.

## Materials and methods

All reagents used to perform the following experiments were quality products from companies such as Sigma Aldrich (St. Louis, MO, USA) and Merck (Whitehouse Station, NJ, USA). All synthetic peptides were purchased from Chinese Peptide Company (Beijing, China).

### *In vitro* cleavage and peptide identification

Identification of the cleavage fragment, was performed as previously described^23^. Briefly, COL6 purified from human placenta (cat. No. ab7538, Abcam Cambridge, UK), was cleaved with pro-MMP2 or pro-MMP-9 (cat. no. 444213; 444231, Calbiochem, Merck, NJ, USA). The proteases were activated by 20 µL 1 mM 4-aminophenylmeruric acetate in dimethyl sufoxide and incubated for 3 hours in 37 °C. The purchased COL6 was dissolved in 0.01% sodium acid and 0.6% acetic acid. To remove proteins below 10 kDA and change the buffer to a neutral pH suitable for cleavage analysis, the purified human COL6 was filtered through a Microcon filter (Merck Millipore, cat. MRCPRT010, Billerica, MA, USA). Subsequently, COL6 was re-suspended to 1 mg/mL and diluted in the ratio 1:3 in MMP cleavage buffer. Hereafter, 1 µg of the MMPs were mixed with 100 µg COL6 in MMP-buffer (100 mM Tris-HCl, 100 mM NaCl, 10 mM CaCl_2_, 2 mM Zn acetate, pH 8.0). As controls, MMP buffer with wither COL6 or MMPs were mixed alone. Cleavages were performed for 24 H at 37 °C, and subsequently stopped by 50 µM EDTA. These fragments were then identified using liquid chromatography (LC) coupled to electrospray ionization (ESI) tandem mass spectrometry (LC-MS/MS). LC was performed by the nanoACQUITY UPLC BEH C18 column (Waters, Milford, MA, USA) using a formic acid/acetonitril gradient, while MS and MS/MS were performed on a Synapt High Definition Mass Spectrometry quadruple time of flight MS (QUAD-TOF; Waters, Milford, MA, USA), with an acquisition range of 350–1600 m/z in MS and 50–2000 m/z, in MS/MS. The six first amino acids from the C-terminal of the cleavage site from COL6, was identified as a neo-epitope generated by the selected proteases and chosen for immunization.

The same cleavage experiment was performed and used to assess the specificity in the C6Mα3 assay.

### Generation of monoclonal antibodies

The generated antibody recognizes a 10 amino acid sequence from the C-terminal generated by cleavage between residues 2288 and 2289 of the α3 chain of COL6 (2279′GPKGGIGNRG.’2288).

At present, only the human protein sequence of COL6 α3 has been annotated. However, when performing a pblast to discover the predicted sequences of mus musculus, rat rattus, sus scrofa and bos Taurus, we found one mismatch for mus musculus (GPKG**S**IGNRG), rat rattus (GPKGG**T**GNRG) and bos taurus (GPKG**S**IGNRG), while two mismatches were found for sus scrofa (GPKGG**L**G**S**RG) compared to the human annotated sequence. The sequences therefore seems to be very alike, but since the antibody targets the C-terminal from the cleavage site the mismatches are at position three and five for sus scrofa, position five for rat rattus, position six for mus musculus and bos taurus. These changes in the amino acid sequence may be crucial for the specificity of the monoconal antibody.

The immunization was performed by subcutaneous injection of 200 uL emulsified antigen and 50 ug immunogenic peptide (KLH-CGG-GPKGGIGNRG) in 4–6 weeks old Balb/C mice using Freund’s incomplete adjuvant. Immunizations were repeated every 2^nd^ week until stable serum antibody titer levels were reached. The mouse with the highest serum titer was selected for fusion and rested for a month. Subsequently, the mouse was boosted intravenously with 50 ug immunogenic peptide in 100 uL 0.9% NaCl solution three days before isolation of the spleen for cell fusion. Hybridoma cells were produced, by fusion of the mouse spleen cells with SP2/0 myeloma cells as described by Gefter *et al*.^24^. By using the semi-solid medium method, hybridoma cells were cultured. Clones were subsequently plated into 96-well microtiter plates for further growth and the limiting dilution method was applied to promote monoclonal growth. A competitive ELISA was performed on streptavidin-coated plates for screening of supernatant reactivity. Biotin-GPKGGIGNRG was used for screening peptide, while the selection peptide GPKGGIGNRG was used for further test of specificity of clones. The supernatant produced by hybridoma cells were purified using HiTrap affinity columns (GE Healthcare Life Science, Little Chalfront, Buckinghamshire, UK) according to manufacturer’s instructions.

### C6Mα3 ELISA methodology

The C6Mα3 competitive ELISA procedure: 96-well streptavidin-coated ELISA plates (Roche, cat. 11940279) were coated with 2.5 ng/mL biotinylated peptide Biotin-GPKGGIGNRG dissolved in assay buffer (25 mM TBS-BTB 2 g. NaCl/L, pH 8.0), 100 µL/well and incubated for 30 min at 20 °C in the dark with 300 rpm shaking. Subsequently, plates were washed five times in washing buffer (20 mM TRIS, 50 mM NaCl, pH 7.2). Subsequently, 20 µL of standard peptide or sample were added to appropriate wells, followed by 100 µL of 10.67 ng/mL monoclonal antibody solution. The plates were incubated for 1 h at 20 °C with shaking, and subsequently washed in washing buffer. Thereafter, 100 µL of horseradish peroxidase (HRP) conjugated rabbit anti-mouse antibody (Jackson ImmunoResearch, cat. 315–035–045, West Grove, Pennsylvania, USA) were added in a 1:3000 solution to the wells, and incubated for 1 h at 20 °C with shaking, and washed five times in washing buffer. Finally, 100 µL 3,3′,5,5-tetramethylbenzinidine (TMB) (Kem-En-Tec cat. 438OH) was added, and incubated for 15 min at 20 °C. To stop the enzyme reaction of TMB, 100 µL of stopping solution (1% H_2_SO_4_) was added. The plate was analyzed by an ELISA reader at 450 nm with 650 nm as reference (Molecular Devices, VersaMax, CA, USA). A standard curve was performed by serial dilution of the selection peptide and plotted using a 4-parametric mathematical fit model. Standard concentrations were 0, 0.39, 0.78, 1.56, 3.13, 6.25, 12.5, and 25 ng/mL. Each plate included five kit controls to monitor intra- and inter-assay variation. All samples were measured within the range of the assay, from lower limit of measurement range (LLMR) and to upper limit of measurement range (ULMR). All samples below LLMR were reported as the value of LLMR.

### Technical evaluation

To assess linearity, a two-fold dilution of four healthy human serum samples was used. The linearity was calculated as a percentage of recovery of the undiluted sample. The lower limit of detection (LLOD) was estimated from 21 determinations of the lowest standard (buffer). LLOD was calculated as mean − 3* standard deviation (SD). Upper limit of detection (ULOD) was determined as the mean ± 3*SD of 10 measurements of Standard A. The intra- and inter-assay variations were determined by 10 independent runs of five quality controls (QC) and two kit controls run in double determinations. Each run consisted of two replicas of double determinations of the samples. LLMR and ULMR were calculated based on the 10 standard curves from the runs performed to assess intra- and inter-assay variation. To test the analyte stability, three healthy human serum samples were stored at either 4 or 20 °C for 2, 4 and 24 h respectively. Stability of the samples were evaluated by calculating the percentage variation in proportion to the sample kept at −20 °C (0 hour sample). Furthermore, the analyte stability was determined for three healthy human serum samples, exposed to four freeze and thaw cycles. To assess the stability of the analyte, the percentage of recovery was calculated using as reference the sample that underwent only one freeze/thaw cycle. The antibody specificity to the standard peptide was studied. The specificity study included a nonsense peptide (AYAKYADFSI), an elongated peptide (GPKGGIGNRGP), and a nonsense coater (Biotin-NTAYAKYADFSISP), used for determination of cross-reactivity. Accuracy was measured in three healthy human serum samples. The samples were spiked with known concentrations of standard peptide and spiking recovery was determined by calculating the percentage recovery of serum spiked in buffer. To test whether the assay interfered with common substances in blood, a interference panel of human samples spiked with biotin (low = 30 ng/ml, high = 90 ng/ml), hemoglobin (low = 0.155 mM, high = 0.310 mM), or lipids (low = 4.83 mM, high = 10.98 mM) was measured. Whether these substances showed to interfere with the assay measurements, was calculated as the percentage recovery using as reference the analyte in non-spiked human serum. To define the standard concentration of C6Mα3, the normal range was determined by analyzing serum from 32 healthy humans. Native reactivity was tested in different biological materials such as human serum, heparin- and EDTA plasma.

### Biological validation of C6Mα3

The newly developed biomarker C6Mα3 was measured in three different cohorts obtained from Bispebjerg Hospital, Odense University Hospital, and San Matteo Hospital of Pavia (Italy). Informed consent and approval by the local Ethics Committee were obtained before sample collection and the studies were performed in compliance with the Helsinki Declaration of 1975.

Cohort 1 is a cross sectional cohort including patients diagnosed with UC. Serum samples (n = 58) were collected at Odense University hospital, Odense, Denmark. All patients filed informed consent and the study was approved by the local ethical committee (Ethics committee of Southern Denmark, Odense, Denmark, approval no. S-20070072). Cohort 2 is a cross sectional cohort including patients diagnosed with CD. Serum samples (n = 44) were collected at San Matteo Hospital Foundation in Pavia, Italy. All patients filed informed consent and the study was approved by the local ethical committee (Ethics committee of the Fondazione IRCCS San Matteo Hospital in Pabia, Italy, protocol number 20100039131, approval no. E_20100039131). Cohort 3 included patients diagnosed with colorectal cancer (stage I-IV). Serum samples (n = 39) were collected prior to resection at Bispebjerg Hospital, Copenhagen, Denmark. All patients filed informed consent and the study was approved by the local ethical committee (The Regional Committee of Copenhagen, Denmark; approval no. H-1-2014-048). There were obtained informed written consent from all subject in the three individual cohorts. Tumor staging was evaluated according to the Union for International Cancer Control classification system. Serum from healthy controls (n = 32) was obtained from a commercial vendor (Valley BioMedical, Winchester, VA, USA) who, according to manufacturer’s information, obtained informed consent. Samples from all three sites were collected, processed, and stored in a similar fashion until analysis. Demographic data from patients are shown in Table 1.

**Table 1:** Clinical characteristics.

Clinical parameter	Crohn’s disease (n = 44)	Ulcerative colitis (n = 58)	Colon cancer (n = 39)	Healthy controls (n = 32)
Age, mean (SD)	35.9 (11.68)	37.5 (14.55)	70.7 (13.04)	43 (14.58)
Gender (% males)	18 (41%)	32 (55%)	20 (51%)	24 (75%)
BMI	21.2 (3.09)	25.5 (4.12)	26.6 (4.51)	—
C6Mα3, mean (SD)	2.47 (1.45)	2.17 (0.68)	1.52 (0.79)	0.63 (0.49)

### Ethical statement

Production of monoclonal antibodies performed in mice was approved by the National Authority (The Animal Experiments Inspectorate) under approval number 2013-15-2934-00956. All animals were treated according to the guidelines for animal welfare.

### Statistical analysis

Baseline characteristics of the cohorts are presented as a number and percentage for categorical variables and mean (standard deviation) for continuous variables. Statistical differences for variables between healthy controls and diseased individuals were assessed using a t-test (parametric) for all cohorts. Results are shown as Scatter Plots with mean value and standard error of mean (SEM). The diagnostic power of C6Mα3 was investigated by the area under the receiver operating characteristics (AUROC) curve. For all statistical analysis performed, a p-value below 0.05 was considered significant. Statistical analysis and graphs were performed using GraphPad Prism version 7 (GraphPad Software, Inc., CA, USA).

## Results

### Generation of monoclonal antibodies

The most optimal antibody producing hybridomas were selected by screening the supernatants. An indirect competitive ELISA was used to screen for reactivity against standard peptide and native material. Biotin-GPKGGIGNRG was used as screening peptide. Based on the measured reactivity, the antibody clone NB140-8A5 was selected.

### Technical evaluation

**Figure 1:**
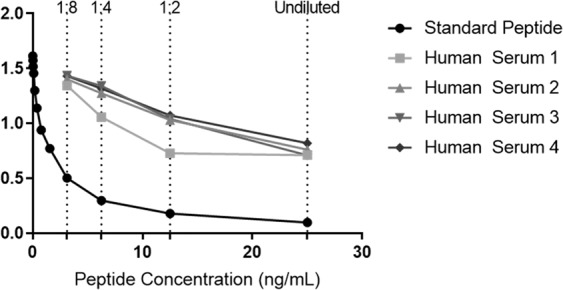
Assay linearity. Inhibition curves for the standard peptide and native material (human serum). The standard peptide was two-fold diluted starting from 25 ng/mL. The samples were tested undiluted and up to eight-fold diluted as indicated. The data are presented as OD (450–650 nm), as a function of peptide concentration. OD = Optical density.

A compete technical evaluation was performed to evaluate the newly developed C6Mα3 ELISA assay. A summary of the technical validation results can be found in Table 2. The measurement range from LLMR to ULMR was 0.219–8.6 ng/mL. The mean intra- and inter-assay variation based on 10 independent assay runs yielded a 8 and 14% variation, respectively. Linearity of the human samples was assessed from undiluted to a 8-fold dilution for human serum (Fig. 1). The analyte stability was acceptable for both 2–5 times freeze/thaw cycles and for prolonged storage of human serum samples. Spiking of standard peptide in human serum resulted in a mean recovery of 54%. Neither high levels of biotin, hemoglobin or lipids interfered with the levels of C6Mα3 in human serum. the mean normal range for C6Mα3 in serum from 32-healthy donors was 0.31 ng/mL.

**Table 2:** C6Mα3 ELISA Technical Validation Data.

Technical validation	C6Mα3
IC50	1.34 ng/mL
Detection range	0.219–8.6 ng/mL
Intra-assay variation^a^	8%
Inter-assay variation^a^	14%
Dilution recovery in serum^a^	96%
Interference biotin, low/high^a^	98%/105%
Interference lipemia, low/high^a^	123%/96%
Interference hemoglobin, low/high^a^	109%/104%
Freeze-thaw stability^a^	105%
Analyte stability^a^	87%
Spiking recovery^a^	54%

^a^Mean recovery percentages are reported.

### Assay specificity

**Figure 2:**
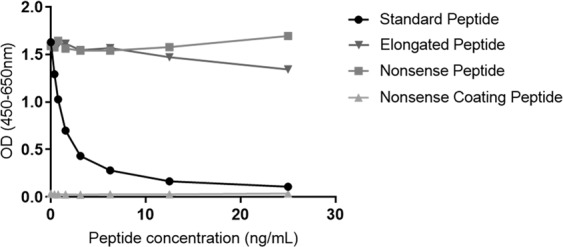
Assay specificity. Inhibition curves for the standard peptide (GPKGGIGNRG), the elongated peptide (GPKGGIGNRGP), a nonsense peptide (AYAKYADFSI) and a nonsense coating peptide (Biotin-NTAYAKYADFSISP). The peptides were two-fold diluted starting from 20 ng/mL. The data are presented as absorbance as a function of peptide concentration. OD = Optical density.

**Figure 3:**
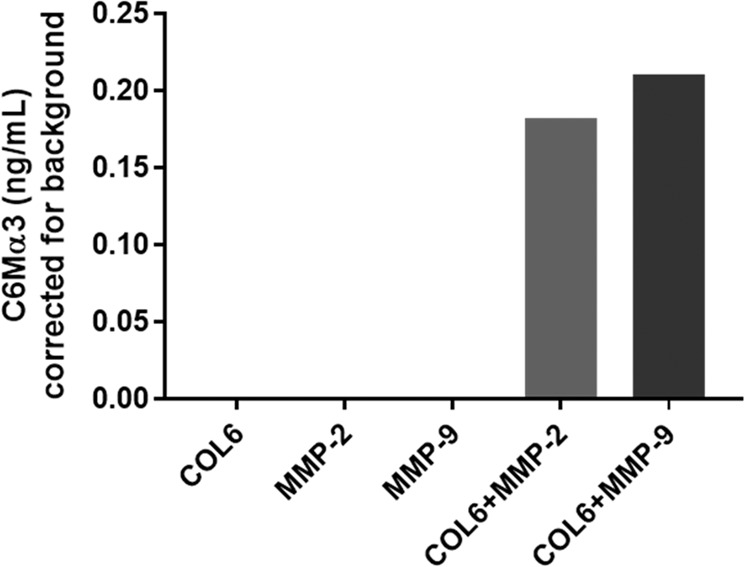
Specificity of the C6Mα3 assay. C6Mα3 fragments after 24 h *in vitro* incubation of human collagen type VI (COL6), with MMP-2 (COL6 + MMP-2) and MMP-9 (COL6 + MMP-9). The negative controls were the enzymes (MMP-2 and MMP-9) without COL6 and the intact COL6 without enzymes. Results are corrected for background.

The analyte detected by the C6Mα3 ELISA was characterized by testing reactivity towards synthetic peptides, to confirm the specificity of the assay. No reactivity was found towards the elongated peptide the nonsense peptide and the nonsense coater (Fig. 2). *In vitro* cleavage experiments also showed that the C6Mα3 assay only detected fragments when COL6 was cleaved by MMP-2 or MMP-9 (Fig. 3).

### Biological and clinical evaluation of the C6Mα3 assay

**Figure 4:**
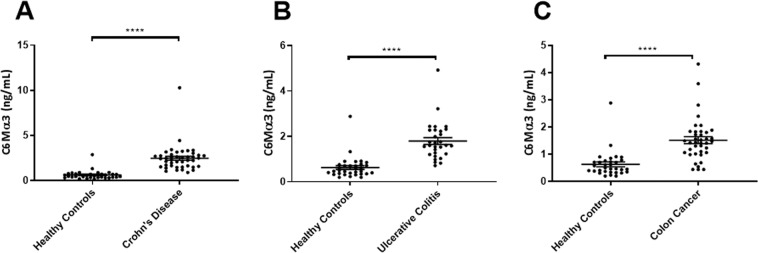
Serum C6Mα3 levels in patients with, (**A**) Crohn’s Disease (CD, n = 44), (**B**) Ulcerative Colitis (UC, n = 58), and (**C**) Colorectal Cancer (CRC, n = 39) compared to healthy controls (n = 32). Data were analyzed using a Mann-Whitney *t* test. Data are presented as Scatter Plots with mean value and standard error of mean (SEM). Significance levels: ****p < 0.0001.

**Figure 5:**
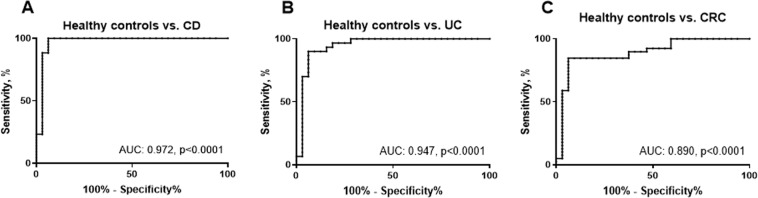
Receiver operating characteristic (ROC) curves. ROC curve analysis were used to evaluate the ability of C6Mα3 to discriminate between; (**A**) Healthy controls and CD patients, (**B**) Healthy controls and UC patients and, (**C**) Healthy controls and CRC patients.

To determine whether C6Mα3 can be used as a marker of gastrointestinal disorders, it was measured in serum from three different cohorts. Here patients with UC (Cohort 1), CD (Cohort 2), CRC (Cohort 3) were compared to healthy controls. The level of C6Mα3 was significantly increased in patients with UC, CD and CRC (all, p < 0.0001, Fig. 4A–C) compared to healthy controls. The diagnostic accuracy (AUROC) of C6Mα3 for patients diagnosed with UC compared to healthy controls was 0.972 (95% CI: 0.925–1.020, p < 0.0001, Fig. 5A), CD compared to healthy controls was 0.947 (95% CI: 0.885–1.009, p < 0.0001, Fig. 5B) and CRC to healthy controls was 0.890 (95% CI: 0.809–0.972, p < 0.0001, Fig. 5C). Thus indicating that C6Mα3 is elevated in patients with gastrointestinal disorders.

## Discussion

In the present study, we developed and characterized a novel specific neo-epitope ELISA detecting a MMP-2- and MMP-9-generated fragment of COL6 α3-chain, named C6Mα3. The main findings of this study were: (i) the assay was technically robust and specific towards the human sequence 2279′GPKGGIGNRG.’2288, (ii) the fragment was detectable in human serum, and (iii) this specific fragment was significantly elevated in human serum from patients with UC, CD, CRC compared to healthy controls with a moderate separation showed. To our knowledge, this is the first study to develop and validate a specific biomarker of MMP-generated COL6 α3-chain and validate this in patients with gastrointestinal disorders.

The C6Mα3 assay was characterized as a technically robust and sensitive assay, with an IC-50 value of 1.34 ng/mL, and a measurement range between 0.2–8.6 ng/mL. All technical tests including dilution recovery, analyte stability, interference and inter- and intra-variation gave results within the acceptable range. The assay specificity test showed that the generated monoclonal antibody was specific towards the MMP-generated cleavage site located between the 2288 and 2289 amino acid in the α3-chain of COL6.

Previously, a fragment COL6α1 generated by MMPs was found to be elevated in serum of patients with CD and was associated with biochemically active disease^25^. This is in line with the findings by Moriggi *et al*. demonstrating that COL6 expression was increased in the inflamed tissue of patients with UC and CD^20^. In addition, COL6 has also been associated with increased integrin expression in IBD and increased lymphocyte homing and influx into the intestinal tissue^20^. Thus, elevated COL6 expression in IBD, represented by increased levels of COL6 α3 chain may be associated with poor outcome by increased tissue inflammation and remodeling during the course of the disease. In addition, elevated levels of COL6 α3 chain fragments in the blood of IBD patients may also be related to the overall state of the mucosa, since COL6 is associated with the integrity of the epithelium^10,20^. In agreement with the present findings, a previous study also reported increased levels of COL6α3 fragments in plasma from patients with CRC^14^. Likewise, a circulating COL6α3 fragment known as endotrophin, has been shown to be associated with survival outcome in patients with metastatic CRC^26^. The COL6 α3 chain has also been found upregulated in the peripheral blood of patients with pancreatic cancer^27^. Altogether this supports a potential for the C6Mα3 biomarker in the diagnostic and prognostic setting for patients with CRC, however the exact applicability should be further evaluted in larger clinical cohorts.

The present findings could indicate that COL6 α3 chain turnover is a common denominator of gastrointestinal disorders. Whether this overlapping pathological mechanism is driven by chronic inflammation, ongoing wound healing and/or fibrosis, individually or combined, deserves further investigations. Future studies should aim to unravel the impact of the degradation of the COL6 α3 chain in terms of disease activity in IBD. As COL6 is closely connected to the basement membrane and the mucosal epithelial cells, the C6Ma3 biomarker could be associated with endoscopic assessment of disease activity for both UC and CD.

Limitations of this study include the assessment of C6Mα3 in relatively small cohorts and in a cross-sectional design. Moreover, we only had limited clinical information on the patients, and no validation cohorts. Assessment of C6Mα3 in larger longitudinal studies are needed to fully evaluate the potential for C6Mα3 as a diagnostic and/or prognostic marker for gastrointestinal disorders, and determine whether the biomarker is able to separate the individual gastrointestinal disorders. One more limitation, is that COL6 is not unique to the gastrointestinal tract, but may also be present in other soft tissues in the body^28–30^. This biomarker can therefore not be categorized as tissue specific.

## Conclusion

We designed and developed the technically robust assay C6Mα3, which targets a MMP-2 and MMP-9 generated fragment of the α3 chain of COL6. C6Mα3 was elevated in serum from patients with UC, CD and CRC compared to healthy controls. These findings need to be further evaluated in larger studies to elucidate the role of C6Mα3 as a diagnostic and/or prognostic biomarker of gastrointestinal disorders.
